# Genetic and epigenetic alterations on the short arm of chromosome 11 are involved in a majority of sporadic Wilms' tumours

**DOI:** 10.1038/sj.bjc.6603302

**Published:** 2006-08-08

**Authors:** Y Satoh, H Nakadate, T Nakagawachi, K Higashimoto, K Joh, Z Masaki, J Uozumi, Y Kaneko, T Mukai, H Soejima

**Affiliations:** 1 Division of Molecular Biology and Genetics, Department of Biomolecular Sciences, Faculty of Medicine, Saga University, 5-1-1 Nabeshima, Saga 849-8501, Japan; 2 Department of Urology, Faculty of Medicine, Saga University, 5-1-1 Nabeshima, Saga 849-8501, Japan; 3 Department of Pediatrics, Kitasato University Hospital, 1-15-1 Kitasato, Sagamihara, Kanagawa 228-8555, Japan; 4 Department of Surgery, Faculty of Medicine, Saga University, 5-1-1 Nabeshima, Saga 849-8501, Japan; 5 Saitama Cancer Center, Research Institute for Clinical Oncology, 818 Komuro, Ina, Saitama 362-0806, Japan

**Keywords:** Wilms' tumour, genetics, epigenetics, loss of heterozygosity, loss of imprinting, DNA methylation

## Abstract

Wilms' tumour is one of the most common solid tumours of childhood. 11p13 (*WT1* locus) and 11p15.5 (*WT2* locus) are known to have genetic or epigenetic aberrations in these tumours. In Wilms' tumours, mutation of the *Wilms tumour 1* (*WT1*) gene at the *WT1* locus has been reported, and the *WT2* locus, comprising the two independent imprinted domains *IGF2*/*H19* and *KIP2*/*LIT1*, can undergo maternal deletion or alterations associated with imprinting. Although these alterations have been identified in many studies, it is still not clear how frequently combined genetic and epigenetic alterations of these loci are involved in Wilms' tumours or how these alterations occur. To answer both questions, we performed genetic and epigenetic analyses of these loci, together with an additional gene, *CTNNB1*, in 35 sporadic Wilms' tumours. Loss of heterozygosity of 11p15.5 and loss of imprinting of *IGF2* were the most frequent genetic (29%) and epigenetic (40%) alterations in Wilms' tumours, respectively. In total, 83% of the tumours had at least one alteration at 11p15.5 and/or 11p13. One-third of the tumours had alterations at multiple loci. Our results suggest that chromosome 11p is not only genetically but also epigenetically critical for the majority of Wilms' tumours.

Wilms' tumour, also known as nephroblastoma, is one of the most common solid tumours of childhood, accounting for approximately 6% of all childhood malignancies. Chromosomal region 11p13 was first identified as a Wilms' tumour locus, *WT1*, because the region was found to be deleted in Wilms' tumours (Kaneko *et al*, 1981; Huff, 1998; Dome and Coppes, 2002, OMIM 194070). The *Wilms tumour 1* (*WT1*) gene, isolated from the *WT1* locus, was the first causative gene for Wilms' tumour (Call *et al*, 1990; Gessler *et al*, 1990). However, *WT1* aberrations, such as deletions and point mutations, are observed in only approximately 10–20% of Wilms' tumours (Huff, 1998; Nakadate *et al*, 2001). The small number of *WT1* mutations in Wilms' tumours suggests that *WT1* can be inactivated by alterations that would not be detected by mutational analysis (Huff, 1998). On the other hand, although *WT1* mutation is not frequent, *WT1* mutation and *CTNNB1* (*β-catenin*) mutation at 3p21 are significantly correlated with Wilms' tumours (Maiti *et al*, 2000).

Loss of heterozygosity (LOH) of 11p15.5, which is known as the *WT2* locus (OMIM 194071), is observed in Wilms' tumours. LOH occurs on the maternal chromosome, suggesting the involvement of genomic imprinting in Wilms' tumorigenesis. This imprinted region is well characterised, and is divided into two imprinted domains, *IGF2*/*H19* and *KIP2*/*LIT1* (Feinberg, 2000). It has been reported that *IGF2* and *H19* within the *IGF2*/*H19* domain are expressed abnormally in Wilms' tumours. *IGF2* encodes an embryonal growth factor and is transcribed exclusively from the paternal allele (Reik and Murrell, 2000), and *H19* is a noncoding RNA with reciprocal transcription from the maternal allele. In Wilms' tumours, abnormally high levels of *IGF2* mRNA and loss of imprinting (LOI) of *IGF2*, allowing both paternal and maternal alleles to be transcribed, have been observed (Reeve *et al*, 1985; Ogawa *et al*, 1993; Rainier *et al*, 1993). LOI of *IGF2* is always accompanied by *H19* biallelic hypermethylation, leading to inactivation of *H19* (in the normal situation, the region upstream of *H19* is methylated only on the paternal allele) (Moulton *et al*, 1994; Steenman *et al*, 1994). Demethylation of *DMR-LIT1*, an imprinting control region (ICR) of the *KIP2*/*LIT1* domain, occurs in half of all patients with Beckwith–Wiedemann syndrome (BWS) (OMIM 130650), which predisposes patients to embryonal tumours, and in a variety of adult tumours. The *p57*^*KIP2*^ (*KIP2*)/*CDKN1C* gene within the *KIP2*/*LIT1* domain, which is expressed predominantly from the maternal allele, encodes a cyclin-dependent kinase inhibitor and is a putative tumour suppressor. In several adult tumours, *KIP2* expression is epigenetically reduced (Shin *et al*, 2000; Kikuchi *et al*, 2002; Li *et al*, 2002; Soejima *et al*, 2004). However, *KIP2* expression has been found to be reduced in Wilms' tumours in some studies, but not in others (Chung *et al*, 1996; Hatada *et al*, 1996; Thompson *et al*, 1996; O'Keefe *et al*, 1997; Taniguchi *et al*, 1997; Soejima *et al*, 1998).

Although several genes involved in Wilms' tumour have been identified, as described above, the alteration frequencies of these genes (loci) and how many loci are altered in the tumour are still unknown. To investigate this, we comprehensively investigated genetic and epigenetic alterations of three loci – *WT1* (11p13), *WT2* (11p15.5), and *CTNNB1* (3p21) — in 35 sporadic Wilms' tumours (Figure 1). Our data indicate that genetic and/or epigenetic alterations of genes at these loci, especially *WT1* and *WT2*, is involved in the majority of Wilms' tumours, and that alterations of multiple loci occur in one-third of tumours. These findings suggest that genetic and epigenetic alterations on the short arm of chromosome 11 play an important role in Wilms' tumorigenesis.

## MATERIALS AND METHODS

### DNA and RNA

In all, 35 tissue samples from sporadic Wilms' tumours and five tissue samples from mid-gestational fetal kidneys were obtained from Saitama Cancer Center Hospital (Saitama, Japan) and the fetal tissue bank at the University of Washington, (WA, USA), respectively. Genomic DNA and total RNA were extracted with a QIAamp DNA mini kit (Qiagen, Hilden, Germany) and Isogen (Nippon Gene, Tokyo, Japan), respectively.

### Mutation and LOH analyses

Genetic analyses of *WT1* were carried out as previously described (Nakadate *et al*, 1999, 2001). Briefly, *WT1* loci were screened for mutations by single-strand conformation polymorphism (SSCP) analysis of all exons and splice-donor/acceptor sites. When an aberrant band was identified by SSCP, the band was excised and sequenced. Loss of heterozygosity was also analysed using polymorphic DNA markers as follows to compare tumour tissue with adjacent normal tissue or peripheral blood: *D11S16*, *D11S325*, *PAX6*, *D11S324*, *WT1*, and *CAT* for 11p13; and *D11S12*, *D11S922*, *D11S932*, *IGF2*, *INS*, and *TH* for 11p15.5. Mutations in exon 3 of the *CTNNB1* gene were investigated by PCR-directed sequencing as previously described (Satoh *et al*, 2003).

### Quantitative real-time reverse transcription (RT)–polymerase chain reaction (PCR)

Total RNA (500 ng) was treated with RNase-free DNase I (Roche, Basel, Switzerland) and reverse-transcribed with ReverTra Ace reverse transcriptase (Toyobo, Japan) and random primers (TaKaRa, Japan). Quantitative real-time RT–PCR was performed with the LightCycler™ system (Roche) according to the manufacturer's protocol. The expression of *WT1* was normalised with that of *β-actin*, as previously described (Satoh *et al*, 2003). The average *WT1* expression of four mid-gestational fetal kidneys was employed as a standard. All experiments were performed in triplicate.

### Allele-specific expression of *IGF2*

Genotyping of *IGF2* was performed by PCR-restriction fragment length polymorphism (RFLP) using a polymorphic *Hae*III (*Apa*I or *Ava*II) site in exon 9, as previously described (Soejima and Yun, 1998). To eliminate genomic DNA contamination, the RNA-specific product (1120 bp) was amplified by using an exon connection primer pair (5′-TCCTGGAGACGTACTGTGCTA-3′ and 5′-GGTCGTGCCAATTACATTTCA-3′). To further eliminate contaminating DNA, the RNA-specific product was excised from 1% agarose gel after electrophoresis and purified. Then, the purified product was subjected to nested PCR and RFLP analysis with *Hae*III (Yun *et al*, 1999).

### Methylation analyses

Combined bisulphite restriction analyses (COBRA) using the hot-stop method were employed to determine the extent of methylation at the differentially methylated region (DMR) of the *H19* promoter (*H19-pro-DMR*), *DMR-LIT1*, and *WT1* promoter. Although an ICR of the *IGF2*/*H19* domain exists between 2 and 5 kb upstream of the *H19* gene, we analysed *H19-pro-DMR* because *IGF2* LOI uniformly correlates with hypermethylation of *H19-pro-DMR* (Moulton *et al*, 1994; Steenman *et al*, 1994). The primer pairs and restriction endonucleases used were as follows: 5′-GGGAGGGTTTTGTTTTGATTGGT-3′, 5′-ACTCTCCTCCAACACCCCATCTTC-3′, and *Hin*fI for *H19-pro-DMR*; and 5′-CGTATTCGATTTTGTTCGGATTT-3′; 5′-ACTACCCTCAACTTCCCAAAACT-3′, and *Hin*fI for the *WT1* promoter. For several samples, methylation of *H19-pro-DMR* was confirmed by using hot-stop COBRA for a region immediately downstream of CTCF binding site 6 (CTCF6) in *H19*-*DMR.* The primer pairs and the restriction endonuclease used were 5′-GAGTTYGGGGGTTTTTGTATAGT-3′, 5′-TAAATAATACCCRACCTAAAAATCTAA-3′, and *Mlu*I. *DMR-LIT1* was analysed as previously described (Soejima *et al*, 2004). The hot-stop COBRA products were separated by 7.5% polyacrylamide gel electrophoresis (PAGE) and quantified with BAS2000 (Fujifilm, Japan). All experiments were performed three times independently.

## RESULTS

### Genetic and epigenetic alteration of the *IGF2/H19* imprinted domain at 11p15.5

Of 35 tumours, 10 (29%) showed LOH of 11p15 and 25 showed retention of heterozygosity (ROH) at this locus (Tables 1 and 2). 11p15.5 LOH involved loss of both the *IGF2*/*H19* and *KIP2*/*LIT1* imprinted domains. Although three tumours (#33, #34, and #35) were not informative for polymorphisms, these were considered to have undergone LOH because of hypermethylation of *H19-pro-DMR* and hypomethylation of *DMR-LIT1*, indicating loss of the maternal chromosomal region. For another three tumours that were not informative for polymorphisms (#6, #8, and #10), methylation of *H19-pro-DMR* and *DMR-LIT1* was maintained, so they were considered to show ROH.

**Figure 2:**
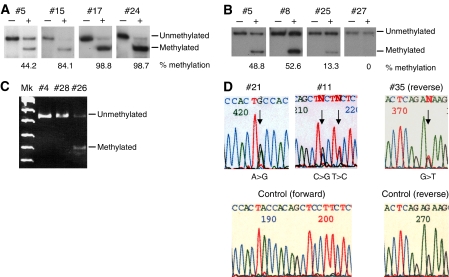
Representative results of this study. (**A**) Methylation analysis of the *H19-pro-DMR* by hot-stop COBRA. Tumour #5 showed normal methylation, whereas #15, #17, and #24 showed hypermethylation. −: not digested by *Hin*fI, +: digested by *Hin*fI. (**B**) Methylation analysis of *DMR-LIT1* by hot-stop COBRA. #5 and #8 showed normal methylation, whereas #25, and #27 showed hypo- or demethylation. −: not digested by *Acc*II, +: digested by *Acc*II. (**C**) Methylation analysis of the *WT1* promoter region by COBRA. #4 and #28 showed no methylation, whereas #26 showed methylation. (**D**) Mutation analysis of *CTNNB1*. Arrows indicate bases that were mutated. Control sequences are shown below.

We examined allelic expression of *IGF2* to screen for epigenetic alterations of the *IGF2*/*H19* imprinted domain. Genotyping revealed that eight tumours (#1–4, #11–14) were heterozygous for polymorphism in *IGF2* exon 9. Reverse transcription–PCR revealed that three of these (#12–14) expressed *IGF2* biallelicaly, that is, LOI had occurred (Table 1). We also examined the methylation status of *H19-pro-DMR* because *IGF2* LOI uniformly correlates with biallelic hypermethylation of *H19-pro-DMR* (Moulton *et al*, 1994; Steenman *et al*, 1994). Five normal mid-gestational fetal kidneys were used as controls for the methylation status of *H19-pro-DMR*. The average percentage methylation of the fetal kidneys was 42.5±8.4% (data not shown), and we defined methylation of more than the average of the fetal kidneys+2 s.d. as hypermethylation. The total number of tumours showing *H19-pro-DMR* hypermethylation was 21, comprising 11 with ROH and 10 with LOH (Table 1, Figure 2A). Because LOH occurs with the maternal chromosome, only the methylated paternal chromosome remains in LOH tumour cells, resulting in hypermethylation. Thus, biallelic hypermethylation leading to *IGF2* LOI occurred in 11 tumours with ROH. Indeed, all three tumours (#12, #13, #14) that were heterozygous for the polymorphism and showed biallelic expression also showed hypermethylation (data not shown). Furthermore, representative samples with hypermethylation at *H19-pro-DMR* also underwent hypermethylation at *H19-DMR* CTCF6 (data not shown). A total of 14 out of 35 tumours (40%) had LOI (Tables 1 and 2); and LOI occurred in 56% of ROH tumours (14 out of 25).

### Epigenetic alteration of the *KIP2/LIT1* imprinted domain at 11p15.5

We investigated methylation of *DMR-LIT1* in the *KIP2*/*LIT1* imprinted domain (Tables 1 and 2, Figure 2B) relative to the average percentage methylation in fetal kidneys, which was 44.3±7.5% (data not shown). We defined methylation of less than the average of the fetal kidneys – 2 s.d. as hypomethylation. Although 12 tumours, eight with LOH and four with ROH, showed hypomethylation, the four with ROH had biallelic hypomethylation because maternal *DMR-LIT1* is normally methylated. In spite of LOH, two tumours (#26 and #32) did not have a methylation level that was less than the average for the fetal kidneys – 2 s.d., but still had a low level of methylation (29.7 and 39.8%). These findings might be due to contamination with nontumour cells.

We also investigated expression and promoter methylation of *KIP2*, because this imprinted gene is a putative tumour suppressor gene, but no somatic mutation has been found in tumours to date. *KIP2* expression varied from zero to approximately 800% of that of the control fetal kidneys, and the promoter region was not methylated in any sample (data not shown). In addition, there was no correlation between *KIP2* expression and *DMR-LIT1* methylation.

A total of 25 (71%) tumours showed alteration of *IGF2*/*H19* or *KIP2*/*LIT1* or both of the domains, of which 10 showed LOH, 11 showed *IGF2* LOI only, one showed *DMR-LIT1* hypomethylation only, and three showed both *IGF2* LOI and *DMR-LIT1* hypomethylation (Table 1).

### Genetic and epigenetic alteration of *WT1* at 11p13

A total of 20 tumours were informative for polymorphisms on 11p13: 12 of these had preserved heterozygosity and five (25%) showed 11p13 LOH, and these had concurrent 11p15.5 LOH, indicating a large LOH region (more than 30 Mb) in the short arm of chromosome 11 (Tables 1 and 2). *WT1* gene mutation was also examined as a genetic alteration. Only three tumours had homozygous deletion of *WT1*, as previously described (Nakadate *et al*, 1999; Watanabe *et al*, 2006).

As epigenetic alterations, the expression and promoter methylation of *WT1* were examined. We determined the quantity of *WT1* expression normalised with *β-actin* expression. We defined expression of less than 10% of that of the control fetal kidneys as a significant reduction, and found seven tumours with such a reduction (Table 1). Two tumours (#9 and #10) expressed a certain level of *WT1* in spite of a homozygous deletion, which might be due to contamination with nontumour cells. Excluding tumours with homozygous deletions, six tumours (17%) had a reduction in *WT1* expression (Tables 1 and 2). Methylation analysis, however, revealed that only one tumour (#26) had promoter methylation, as previously described (Table 1 and Figure 2C) (Satoh *et al*, 2003). Promoter methylation was not found in any other tumours with reduction in *WT1* expression.

In summary, genetic alterations of *WT1* such as LOH or *WT1* homozygous deletion were found in a total of eight tumours, and epigenetic alterations (i.e. reduction of *WT1* expression) were found in six (Table 2).

### *CTNNB1* mutation

We found four missense mutations of the *CTNNB1* gene in three tumours: Pro44Ala (CCT to GCT) and Ser45Pro (TCT to CCT) in #11, Thr41Ala (ACC to GCC) in #21, and Ser45Tyr (TCT to TAT) in #35 (Tables 1 and 2, Figure 2D). The tumours with *CTNNB1* mutation had concurrent *WT1* homozygous deletion and *DMR*-*LIT1* hypomethylation*, IGF2* LOI, and 11p15.5 LOH, respectively.

## DISCUSSION

In this study, we investigated genetic and epigenetic alterations of three loci that are thought to be involved in Wilms' tumour: the *WT2* locus (11p15. 5) including the *IGF2*/*H19* and the *KIP2*/*LIT1* imprinted domains, the *WT1* locus (11p13) including the *WT1* gene, and 3p21 locus including the *CTNNB1* gene. Loss of heterozygosity of 11p15.5 was the most frequent genetic alteration (29%), and *IGF2* LOI was the most frequent epigenetic alteration (40%) (Table 2). In ROH tumours only, *IGF2* LOI frequency occurred in approximately 56% of cases (14/25). The data were consistent with the results of previous reports (Ogawa *et al*, 1993; Rainier *et al*, 1993; Steenman *et al*, 1994; Moulton *et al*, 1994, Yuan *et al*, 2005). It is intriguing that three tumours (#23–25) showed alterations of both *IGF2*/*H19* and *KIP2*/*LIT1* imprinted domains, because each domain is independently regulated, and BWS with both alterations is very rare (DeBaun *et al*, 2002). Furthermore, #25 had a reduction of *WT1* expression. The data suggest that 11p is epigenetically unstable in Wilms' tumours. With regard to the number of altered loci, 18 tumours (51%) showed alteration at only one locus and 11 (31%) showed alterations at multiple loci (Table 3). Six (18%) tumours did not show any alteration. Thus, 83% (29 out of 35) of Wilms' tumours had alterations at one or more of the three loci. Furthermore, no tumour had mutation of *CTNNB1* alone. These results indicate that the alterations observed in Wilms' tumours are concentrated on the short arm of chromosome 11, that is 11p15.5-p13, and that the region is not only genetically but also epigenetically critical for Wilms' tumorigenesis.

**Figure 3:**
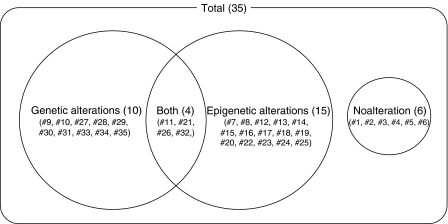
Schematic diagram summarising alterations of the three loci in a total of 35 sporadic Wilms' tumours. Genetic alterations comprise LOH, *WT1* mutation, and *CTNNB1* mutation. Epigenetic alterations comprise *IGF2* LOI, *DMR-LIT1* hypomethylation, and *WT1* reduction.

As shown in Figure 3, there were 10 and 15 tumours, respectively, with only genetic or only epigenetic alterations. Four tumours had both genetic and epigenetic alterations. The average age of patients at diagnosis for tumours with only genetic and only epigenetic alterations was 34.8±33.3 and 46.5±24.1 months, respectively, but there was no significant difference between them.

Because maternal LOH of 11p15.5 is uniformly accompanied by paternal duplication, it results in two paternal copies of the *IGF2* gene and an increase of *IGF2* expression. In addition, *IGF2* LOI is observed in non-neoplastic kidney parenchyma and frequently in early-stage tumours, indicating the importance of *IGF2* in Wilms' tumorigenesis (Moulton *et al*, 1994; Okamoto *et al*, 1997; Yuan *et al*, 2005). However, in a recent study, *IGF2* LOI was not observed in any of 21 Wilms' tumours from Japanese patients (Fukuzawa *et al*, 2004). In that study, the *Hpa*II site near the CTCF6 in *H19*-*DMR*, which is approximately 2 kb upstream from the *H19* transcription initiation site, was used to analyse *IGF2* LOI using real-time PCR. In the present study, we employed RT–PCR–RFLP and hot-stop COBRA for analysis of the methylation of *H19*-*pro*-*DMR*. Further, the results of *H19*-*pro*-*DMR* were confirmed by *H19-DMR* CTCF6 with hot-stop COBRA. Our results clearly show that *IGF2* LOI occurs in Japanese patients with Wilms' tumour. At present, we are not able to explain the discrepancy, but having a small sample size might have influenced the results.

Although *KIP2* expression is epigenetically reduced in several adult tumours (Shin *et al*, 2000; Kikuchi *et al*, 2002; Li *et al*, 2002; Soejima *et al*, 2004), expression levels in Wilms' tumour as measured in previous studies have varied (Chung *et al*, 1996; Hatada *et al*, 1996; Thompson *et al*, 1996; O'Keefe *et al*, 1997; Taniguchi *et al*, 1997; Soejima *et al*, 1998). In the present study, *KIP2* expression also varied, suggesting that at least in Wilms' tumour, *KIP2* may not be involved.

*WT1* gene expression was reduced in six (17%) tumours. It is noteworthy that the frequency of *WT1* reduction in expression is similar to that of *WT1* mutation. *WT1* expression reduction is correlated with predominant stromal histology (Pritchard-Jones *et al*, 1990; Miwa *et al*, 1992). Our tumours comprised one stromal, two triphasic, and three blastemal types. Although the precise histologic composition of tumours in the present study was unknown, whether or not there is a correlation between the *WT1* expression reduction and histology is not clear because the number of tumours was small. Only one tumour (#26) had promoter hypermethylation, as described previously (Satoh *et al*, 2003). Since this tumour also had concurrent 11p13 LOH, ‘two-hit’ inactivation (LOH and methylation) led to a reduction of *WT1* expression. However, methylation was not found in any other tumours with *WT1* expression reduction, thus promoter methylation might not be fundamentally involved in *WT1* transcriptional repression. *WT1* transcriptional regulation is remarkably complex, and our knowledge of it is still quite limited (Englert, 1998). Thus, other unknown mechanisms may be involved in the reduction of *WT1* expression.

A highly significant correlation has been found between *WT1* mutation and *CTNNB1* mutation in Wilms' tumours (Maiti *et al*, 2000). *β*-Catenin, a product of the *CTNNB1* gene, is involved in the regulation of cell adhesion and in signal transduction through the WNT pathway. Abrogation of the WNT pathway by *CTNNB1* mutations, resulting in reduced serine/threonine phosphorylation, has been recognised as playing an important role in the development of many tumours. All *CTNNB1* mutations we found occurred at or near phosphorylation sites. Only one tumour had concurrent homozygous deletion of the *WT1* gene. Whether or not there is a correlation between the gene mutations is not clear because the number of tumours with mutations was too small.

In conclusion, genetic and epigenetic alterations of chromosome 11p play an important role in the majority of Wilms' tumours. There is a possibility that not only the genes investigated in this study but also unidentified genes existing in the region with unknown function also play an important role in Wilms' tumorigenesis. In addition, six tumours did not have any alterations at the three loci studied, suggesting the involvement of genes at other loci. Chromosomes 1p, 4q, 7p, 11q, 14q, 16q, and 17p are also frequently lost in Wilms' tumours, and the *RASSF1A* tumour suppressor is frequently silenced by promoter hypermethylation (Ehrlich *et al*, 2002; Harada *et al*, 2002; Wagner *et al*, 2002; Yuan *et al*, 2005). Identification of a novel gene or genes at these loci and those silenced by epigenetic mechanisms will be helpful to further understand Wilms' tumorigenesis.

## Figures and Tables

**Figure 1:**
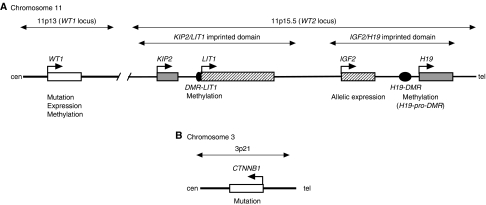
Maps of regions analysed in this study. (**A**) *WT1* locus and *WT2* locus on the short arm of chromosome 11. Representative genes are shown. Broken arrows indicate transcriptional direction. Grey boxes and shaded boxes indicate maternal and paternal expression, respectively. *DMR-LIT1* and *H19*-*DMR* are the ICRs for each domain, respectively. *DMR-LIT1* is differentially methylated on the maternal allele. The *H19*-*DMR* and *H19* promoter are differentially methylated on the paternal allele. The items examined in this study are shown below each gene or DMR. (**B**) *CTNNB1* locus. Maps are not to scale.

**Table 1:** Genetic or epigenetic alterations in Wilms' tumours

	**11p15.5 (*WT2* locus)**			**11p13 (*WT1* locus)**			**3p21**	
**Sample no.**	**11p15.5 LOH**	***IGF2* LOI**	***DMR-LIT1***	**11p13 LOH**	***WT1* mutation**	***WT1* express.^a^**	***CTNNB1* mutation**	**Alteration type^b^**
1	−	ROI (p)	Normal	−	−	128.1	−	None
2	−	ROI (p)	Normal	ND	−	114.3	−	None
3	−	ROI (p)	Normal	−	−	128.6	−	None
4	−	ROI (p)	Normal	ND	−	385.7	−	None
5	−	ROI (m)	Normal	−	−	57.1	−	None
6	(−)^c^	ROI (m)	Normal	ND	−	385.7	−	None
7	−	ROI (m)	Normal	−	−	**0.0**	−	**E**
8	(−)^c^	ROI (m)	Normal	ND	−	**0.0**	−	**E**
9	−	ROI (m)	Normal	**HD**	**HD**	28.6	−	**G**
10	(−)^c^	ROI (m)	Normal	**HD**	**HD**	28.6	−	**G**
11	−	ROI (p)	**Hypo**	**HD**	**HD**	**0.0**	**Pro44Ala Ser45Pro**	**G, G, E**
12	−	**LOI (p)**	Normal	−	−	14.3	−	**E**
13	−	**LOI (p)**	Normal	−	−	85.7	−	**E**
14	−	**LOI (p)**	Normal	−	−	28.6	−	**E**
15	−	**LOI (m)**	Normal	−	−	57.1	−	**E**
16	−	**LOI (m)**	Normal	−	−	114.3	−	**E**
17	−	**LOI (m)**	Normal	−	−	442.9	−	**E**
18	−	**LOI (m)**	Normal	NI	−	28.6	−	**E**
19	−	**LOI (m)**	Normal	NI	−	85.7	−	**E**
20	−	**LOI (m)**	Normal	NI	−	1557.1	−	**E**
21	−	**LOI (m)**	Normal	−	−	157.1	**Thr41Ala**	**G, E**
22	−	**LOI (m)**	Normal	−	−	**0.8**	−	**E, E**
23	−	**LOI (m)**	**Hypo**	ND	−	142.9	−	**E**
24	−	**LOI (m)**	**Hypo**	NI	−	171.4	−	**E**
25	−	**LOI (m)**	**Hypo**	ND	−	**0.6**	−	**E, E**
26	+	Hyper	Normal	+	−	**1.2** d	−	**G, G, E**
27	+	Hyper	Hypo	+	−	14.3	−	**G, G**
28	+	Hyper	Hypo	+	−	28.6	−	**G, G**
29	+	Hyper	Hypo	+	−	85.8	−	**G, G**
30	+	Hyper	Hypo	+	−	228.6	−	**G, G**
31	+	Hyper	Hypo	NI	−	857.1	−	**G**
32	+	Hyper	Normal	ND	−	**5.7**	−	**G, E**
33	(+)^e^	Hyper	Hypo	NI	−	385.7	−	**G**
34	(+)^e^	Hyper	Hypo	NI	−	14.3	−	**G**
35	(+)^e^	Hyper	Hypo	ND	−	100.0	**Ser45Tyr**	**G, G**

Genetic and epigenetic alterations are indicated by blue and red bold, respectively. *IGF2* LOI was examined by RT–PCR–RFLP with *Hae*III polymorphism (p) or methylation analysis of *H19-pro-DMR* (m). Hypermethylation of *H19-pro DMR* in 11p15.5 LOH cases was not indicated by red color because it was due to LOH. *WT1* expression in #11 is not indicated by red color because the reduction of this sample was secondary alteration caused by a genetic alteration, homozygous deletion. LOI=loss of imprinting; hyper=hypermethylation of *H19-pro DMR*; hypo=hypomethylation of *DMR-LIT1*; ND=not done; NI=not informative; HD=homozygous deletion.

a *WT1* expression less than 10% of fetal kidneys is considered epigenetic alteration.

b Genetic alteration and epigenetic alteration are indicated by G and E, respectively. Number of G or E indicates number of altered loci.

c These were considered ROH because methylation of *H19-pro-DMR* and *DMR-LIT1* were maintained.

d This sample showed promoter hypermethylation.

e These were considered LOH because of *H19-pro-DMR* hypermethylation and *DMR-LIT1* hypomethylation.

**Table 2:** Frequency of each genetic or epigenetic alteration in Wilms' tumours

**Locus**	**Alteration**	**Alteration type (genetic (G) or epigenetic (E))**	**Sample number**	**Frequency**
11p15.5 (*WT2* locus)	11p15.5 LOH	G	26–35	10/35 (29%)
	*IGF2* LOI	E	12–25	14/35 (40%)
	*DMR-LIT1* hypomethylation	E	11, 23–25	4/35 (11%)
				
11p13 (*WT1* locus)	11p13 LOH	G	26–30	5/20 (25%)
	*WT1* homozygous deletion	G	9–11	3/35 (9%)
	*WT1* reduction	E	7, 8, 22, 25, 26^a^, 32	6/35 (17%)
				
3p21	*CTNNB1* mutation	G	11, 21, 35	3/35 (9%)

a *WT1* promoter in #26 was hypermethylated.

**Table 3:** Number of altered loci in Wilms' tumour

**Genes and loci**	**One locus**			**Two loci**			**Three loci**	**None**
*WT2* locus (11p15.5)	+			+	+		+	−
*WT1* locus (11p13)		+		+		+	+	−
*CTNNB1* (3p21)			+		+	+	+	−
	14	4	0	7	2	0	2	6

+ Indicates genetic or epigenetic alteration at each locus.

*WT2* locus: 11p15.5 LOH or *IGF2* LOI or *DMR-LIT1* hypomethylation.

*WT1* locus: 11p13 LOH or *WT1* mutation or *WT1* reduction.

*CTNNB1*: mutation.
